# Comparison of the effects of citicoline and vascular rehabilitation capsules on neurotrophic and inflammatory factors in patients with cerebral infarction

**DOI:** 10.5937/jomb0-55775

**Published:** 2025-07-04

**Authors:** Dongyun Wu, Jiajun Pan, Xinhong Fang, Zhongjun Chen

**Affiliations:** 1 The Central Hospital of Shangrao, Department of Neurology, Shangrao, Jiangxi, China; 2 Shangrao Peoples's Hospital, Department of Neurology, Shangrao, Jiangxi, China

**Keywords:** butylphthalide, citicoline, vascular dementia after cerebral infarction, inflammatory response, oxidative stress, butilftalid, citikolin, vaskularna demencija nakon cerebralnog infarkta, inflamatorni odgovor, oksidativni stres

## Abstract

**Background:**

Butylphthalide (BP) is commonly used to treat vascular dementia (VD) following cerebral infarction (CI), but BP alone has limited efficacy. BP in combination with citicoline (COPC) or vascular rehabilitation capsules (VRC) is common in clinical practice, but few studies have compared the differences between these two treatment options.

**Methods:**

Ninety-eight patients with VD after CI who were seen in our hospital from April 2020 to June 2022 were selected as the study population. Among them, 52 patients received BP combined with COPC (BP+COPC group), while the rest 46 received BP combined with VRC (BP+VRC group). Fasting venous blood was drawn from the patients before and after treatment. The levels of neurotrophic factor [nerve growth factor (NGF), neuron-specific enolase (NSE), brain-derived neurotrophic factor (BDNF)], oxidative stress [superoxide dismutase (SOD), malondialdehyde (MDA)], and inflammatory factors [Tumor necrosis factor-a (TNF-a), Interleukin-1 (IL-1b), C-reactive protein (CRP)] were measured. Cognitive and neurological improvements were assessed using the Loewenstein Occupational Therapy Cognitive Assessment (LOTCA) and the National Institute of Health Stroke Scale (NIHSS). In addition, the patient's cerebral hemodynamics were examined by CT.

**Results:**

After treatment, LOTCA increased in both groups and was higher in the BP+COPC group than in the BP+VRC group (P<0.05), while NIHSS decreased in the BP+COPC group than in the BP+VRC group (P<0.05). NSE, MDA, IL-1 , CRP, and TNF-a decreased in both groups after treatment, while NGF, BDNF, and SOD increased, again with more significant changes in the BP+COPC group (P<0.05). In addition, cerebral hemodynamics was more favourable in the BP+COPC group than in the BP+VRC group (P<0.05).

**Conclusions:**

BP combined with COPC has superior improvement in neurologic function in patients with VD after CI.

## Abstract (sr)

**Uvod:**

Butilftalid (BP) se često koristi za lečenje vaskularne demencije (VD) nakon cerebralnog infarkta (CI), ali BP sam po sebi ima ograničenu efikasnost. BP u kombinaciji sa citikolinom (COPC) ili kapsulama vaskularne rehabilitacije (VRC) je uobičajena praksa u kliničkoj terapiji, ali postoji Mali broj studija koje su upoređivale razlike između ova dva tretmana.

**Metode:**

Kao populacija nad kojom će se sprovesti istraživanje izabrano je devedeset osam pacijenata sa VD nakon CI koji su lečeni u našoj bolnici od aprila 2020. do juna 2022. godine. Od njih, 52 pacijenta su primala BP u kombinaciji sa COPC (BP+COPC grupa), dok je ostatak od 46 pacijenata primio BP u kombinaciji sa VRC (BP+VRC grupa). Pacijentima je pre i nakon lečenja uzet uzorak venske krvi na postu. Mereni su nivoi neurotrofičkih faktora [faktora rasta nerava (NGF), enolaze specifične za neurone (NSE), faktora rasta derivisanog iz mozga (BDNF)], oksidativnog stresa [superoksid dismutaze (SOD), malondialdehida (MDA)] i inflamatornih faktora [faktor nekroze tumora-alfa (TNF-a), interleukin-1b (IL-1b), C-reaktivni protein (CRP)]. Kognitivna i neurološka poboljšanja su procenjena korišćenjem Loewensteinove ocene kognitivne terapije (LOTCA) i Skale za procenu moždanog udara Nacionalnog instituta za zdravlje (NIHSS). Takođe, hemodinamika pacijenta je ispitana CT-om.

**Rezultati:**

Nakon lečenja, LOTCA se povećala u obe grupe i bila je viša u BP+COPC grupi nego u BP+VRC grupi (P<0,05), dok je NIHSS opao u BP+COPC grupi više nego u BP+VRC grupi (P<0,05). NSE, MDA, IL-1b, CRP i TNF-a su opali u obe grupe nakon lečenja, dok su NGF, BDNF i SOD porasli, ponovo sa značajnijim promenama u BP+COPC grupi (P<0,05). Takođe, cerebralna hemodina - mika je bila povoljnija u BP+COPC grupi nego u BP+VRC grupi (P<0,05).

**Zaključak:**

BP u kombinaciji sa COPC pokazuje superiorno poboljšanje neurološke funkcije kod pacijenata sa VD nakon CI.

## Introduction

With the aggravation of the ageing population in China, the incidence of cerebrovascular diseases is increasing [1]. Cerebral infarction (CI) is characterised by rapid onset and progression. It is a disease with a high global mortality and disability rate, with a very high incidence in the middle-aged and elderly population [2]. Vascular dementia (VD) is a common sequela of CI, a syndrome of memory, cognitive and behavioural dysfunction [3]. CI-induced inflammatory response and oxidative stress can cause severe damage to the nervous system, which is the leading cause of VD [4]. The current clinical treatment for VD after CI is based on the principles of stroke prevention, improvement of cognitive function and control of psychobehavioural symptoms [5]. In addition to interventions for risk factors, combination drug therapy po sitively affects VD patients after CI [6]. Butyl phthalide (BP) is a new class of anti-cerebral ischemic drug developed in China, which has a strong effect and is effective in treating VD with mild adverse effects and is commonly used in clinical cerebrovascular diseases [7] [8]. In addition, Vascular Rehabilitation Capsules (VRC) effectively treat VD by dilating cerebral blood vessels. In contrast, citicoline (COPC), also commonly used as a treatment for VD, can promote brain cell respiration and improve brain function with particular efficacy [9].

Either BP combined with COPC or BP combined with VRC has been shown to treat VD very well [10] [11]. Still, no studies have compared the effects of the two diphtherapy modalities; therefore, the treatment choice for VD remains controversial. In this study, we will compare the difference in the effectiveness of BP in combination with COPC and VRC to inform future clinical use.

## Materials and methods

### Research subjects

Ninety-eight patients with VD after CI who were treated in our hospital from April 2020 to June 2022 were selected as study subjects, and this study met the requirements of our ethics committee. It was reviewed and approved by Shangrao People’s Hospital. Fifty-two of these patients received BP combined with COPC and were BP+COPC group. The rest of the 46 patients received BP combined with VRC and were seen to be in the BP+VRC group. When comparing the clinical baseline data (age, gender, etc.) between both groups, the difference in basic information between the two groups was not statistically significant (P>0.05). It was seen to be comparable (Table 1).

**Table 1:** Clinical baseline information sheet.

	BP+COPC group<br>(n=52)	BP+VRC group(n=46)	t or χ^2^	P
Age	62.8±4.7	62.2±4.8	0.624	0.534
Gender			0.02	0.888
male/female	29 (55.8)/23 (44.2)	25 (54.4)/21 (45.6)		
Duration of illness (months)	3.6±0.6	3.5±0.9	0.654	0.515
BMI (kg/m^2^)	26.3±3.3	26.8±2.7	0.814	0.417
Smoking			0.208	0.649
yes/no	27 (51.9)/25 (48.1)	26 (56.5)/20 (43.5)		
Drinking			0.027	0.869
yes/no	24 (46.2)/28 (53.8)	22 (47.8)/24 (52.2)		

### Inclusion and exclusion criteria

Inclusion criteria: Our attending neurologist, laboratory, and imaging reports diagnosed patients with VD after CI. All were primary patients. All had complete case data. There was no major oncologylike disease. Patients and families gave informed consent. Exclusion criteria: hepatic or renal dysfunction; congenital cognitive impairment; drug allergy; low adherence; comorbid other autoimmune diseases; inability to receive complete treatment.

### Methods

Both groups took BP (CSPC-NBP Pharma - ceutical Co., Ltd., SFDA Approval No. H20050299) orally before meals, 0.2 g/time, 3 times/d. On this basis, the BP+COPC group received COPC (Qilu Pharmaceutical Co., Ltd., SFDA Approval No. H20020220) orally after meals, 0.2 g/times, 3 times/d. BP+VRC group was supplemented with VRC (Shaanxi Dongtai Pharmaceutical Co., Ltd., SFDA Approval No. Z20050719) orally after meals, 1.8 g/dose, 3 times/d. Patients in both groups were treated continuously for 1 month.

### Sample collection and testing

Fasting venous blood was drawn from patients before and after treatment, respectively, and centrifuged for 12 min (400×g) after 30 min at room temperature, and the supernatant was taken for measurement. ELISA was performed to detect neuron-specific enolase (NSE), brain-derived neurotrophic factor (BDNF), nerve growth factor (NGF), Tumor necrosis factor-α (TNF-α), and Interleukin-1β (IL-1β). A fully automated biochemical analyser detected Creactive protein (CRP). Malondialdehyde (MDA) was tested via the thiobarbituric acid method, and the superoxide dismutase (SOD) levels were assessed via the spectrophotometric method. In addition, CT of the brain was performed on the patients to record regional cerebral blood volume (rCBV), mean transit time (MTT), and regional cerebral blood flow (rCBF).

### Scoring criteria

Cognitive and neurological improvements were assessed before and after treatment according to the Loewenstein Occupational Therapy Cognitive Asses - sment (LOTCA) [12] and the National Institute of Health Stroke Scale (NIHSS) [13]. LOTCA (0-100 points): higher scores indicate higher cognitive function. NIHSS (0–42 points): higher NIHSS scores indicate poorer neurological function.

### Statistical methods

SPSS 23.0 software was used to process the data statistically. The counting data were expressed as (rate) and compared via the chi-square test. The measurement data were represented as (x̄±s) and compared via the t-test, and the paired t-test was used for comparison before and after treatment. P<0.05 was considered a statistically remarkable difference.

## Results

### Cognitive and neurological functions before and after treatment

LOTCA after treatment was higher in the BP+COPC group than in the BP+VRC group, and NIHSS was lower than in the BP+VRC group (P<0.05). LOTCA was higher, and NIHSS was lower in both groups after treatment than before (P<0.05). This result indicated that cognitive and neurological functions were better in the BP+COPC group than in the BP+VRC group after treatment (Table 2).

**Table 2:** Cognitive and neurological functions before and after treatment. Note: Comparison with before treatment #P<0.05.

Groups	LOTCA		NIHSS	
	Before	After	Before	After
BP+COPC group (n=52)	48.4±3.3	67.3±5.1#	21.7±3.6	8.6±3.1#
BP+VRC group (n=46)	48.8±3.6	63.3±4.5#	21.8±3.6	12.0±.8#
t	0.574	4.093	0.137	5.669
P	0.567	<0.001	0.891	<0.001

### Neurotrophic factor levels before and after treatment

After treatment, NSE decreased in both groups but was lower in the BP+COPC group than in the BP+VRC group (P<0.05). On the contrary, NGF and BDNF were elevated, which was higher in the BP+COPC group than in the BP+VRC group (P<0.05). The neurotrophic status was better after BP+COPC group treatment (Table 3).

**Table 3:** Changes in neurotrophic factor levels before and after treatment. Note: Comparison with before treatment #P<0.05.

		BP+COPC group<br>(n=52)	BP+VRC group<br>(n=46)	t	P
NSE (μmol/L)	Before	130.6±19.5	130.7±16.5	0.027	0.978
	After	55.8±9.3#	70.9±11.6#	7.145	<0.001
NGF (ng/mL)	Before	4.9±0.9	4.8±1.1	0.495	0.622
	After	12.5±2.6#	9.2±2.9#	5.94	<0.001
BDNF (pg/mL)	Before	6.3±1.5	6.2±1.3	0.35	0.727
	After	12.6±1.3#	9.2±1.4#	12.46	<0.001

### Hemodynamics before and after treatment

In hemodynamics, we observed no statistically significant difference in comparing cerebral perfusion indexes between the two groups before treatment (P>0.05). After treatment, rCBV and rCBF increased in both groups, while MTT decreased (P<0.05). Among them, rCBV and rCBF were higher in the BP+COPC group than in the BP+VRC group, while MTT was lower than in the BP+VRC group (P<0.05) (Table 4).

**Table 4:** Changes in hemodynamics before and after treatment. Note: Comparison with before treatment #P<0.05.

		BP+COPC group<br>(n=52)	BP+VRC group<br>(n=46)	t	P
rCBV (mL/100 g)	Before	1.3±0.3	1.3±0.2	0.309	0.758
	After	2.1±0.3#	1.8±0.4#	4.163	<0.001
rCBF (mL/100 g/min)	Before	31.2±6.1	32.5±5.3	1.101	0.274
	After	43.1±7.4#	39.3±4.1#	3.046	0.003
MTT (s)	Before	5.1±0.8	5.2±1.0	0.903	0.369
	After	2.7±0.8#	3.3±0.6#	4.184	<0.001

### Inflammatory factors (IFs) and oxidative stress before and after treatment

There was no difference in IL-1β, CRP, TNF-α, MDA, and SOD between groups before treatment (P>0.05). After treatment, CRP, TNF-α, IL-1β, and MDA decreased in both groups but were lower in the BP+COPC group than in the BP+VRC group (P<0.05), whereas SOD increased and was also higher in the BP+COPC group than in BP+VRC group (P<0.05) (Table 5).

**Table 5:** Changes in IFs and oxidative stress before and after treatment. Note: Comparison with before treatment #P<0.05.

		BP+COPC group (n=52)	BP+VRC group (n=46)	t	P
IL-1β (pg/mL)	Before	10.2±3.3	10.6±2.9	0.634	0.528
	After	6.2±1.4#	7.5±1.8#	3.732	<0.001
CRP (mg/L)	Before	24.7±4.6	25.0±4.2	0.319	0.752
	After	8.7±1.7#	11.7±2.0#	8.143	<0.001
TNF-α (pg/mL)	Before	29.4±5.0	28.9±4.4	0.529	0.598
	After	16.3±3.2#	17.8±2.9#	2.419	0.018
MDA (μmol/L)	Before	8.7±2.2	8.8±1.8	0.244	0.808
	After	6.1±1.7#	7.2±1.5#	3.377	0.001
SOD (U/L)	Before	79.4±5.7	79.2±5.3	0.179	0.858
	After	106.9±8.0#	99.8±5.7#	4.999	<0.001

## Discussion

It is well known that VD is one of the extremely common sequelae of CI, and VD patients commonly suffer from varying degrees of neurological impairment and cognitive deficits, which seriously affect their quality of life [14] [15]. It is unclear about the comparative therapeutic efficacy of BP combined with COPC and VRC, respectively, in patients with VD after CI. This study compared the effects of the two treatment modalities to provide accurate guidance and reference for the clinical treatment of such patients. It is evident that BP combined with COPC is more effective in treating patients with VD after CI, and therefore, we recommend this treatment option more.

First, there were remarkable improvements in neurological and cognitive functions in both groups after treatment, which indicated that both treatments had excellent therapeutic effects on VD, and the results were consistent with previous studies [16] [17]. It was seen that the improvement of all findings was better in the BP+COPC group than in the BP+VRC group. In addition, the detection of IFs and oxidative stress in both groups also revealed that IL-1β, CRP, TNF-α, and MDA were lower, and SOD was higher in the BP+COPC group after treatment, which also indicated that BP combined with COPC could be more effectively improve oxidative stress injury in VD patients. Presumably, the reason for this may be due to COPC, a nucleoside derivative with a metabolic agonist effect on brain tissue, which affects the synthesis of lecithin biosynthesis in humans, reduces vascular resistance and increases cerebral blood flow, contributing to the recovery of cerebral circulatory function in VD after CI [18]. Besides, BP can effectively block several pathological processes of brain injury, thus exerting a better anti-ischemic effect and improving cerebral microcirculation and blood flow [19]. It can also improve cerebral blood flow by dilating blood vessels and inhibiting platelet aggregation. It can also reduce free radicals by inhibiting superoxide anion radical production and improving brain tissue energy metabolism [20]. When combined with COPC, BP can repair damaged brain tissue, protect cerebral blood vessels, and promote neurological recovery by different mechanisms, improving all patient functions [21]. VRC usually treats vasculitis, arteriosclerosis, lower extremity vascular occlusion, etc. It is also commonly used for sequelae such as coronary heart disease and cerebral thrombosis. They have also achieved a relatively stable effect in treating CI [22].

However, the disadvantage is that the effect is insignificant and usually requires a large dose and a long time to take [23]. Combined with BP, it is equally effective in treating cerebrovascular and brain tissue damage caused by CI and VD. However, BP+COPC compared to BP+VRC, BP combined with COPC can improve patients’ injury from multiple perspectives while providing a good internal environment for the recovery of vascular tissue. BP combined with VRC, on the other hand, proceeds mainly by improving vascular patency, and its repair process is more homogeneous. Therefore, the therapeutic effect of BP combined with COPC is more significant and comprehensive at the same time. Because of this, the results of the current study showed higher rCBV and rCBF and lower MTT in the BP+COPC group after treatment, indicating that BP combined with COPC is more beneficial in improving cerebral hemodynamics in patients. Indeed, the excellent effect of BP in combination with VRC has been well demonstrated in several previous studies [24] [25], and the results of the present trial similarly demonstrate that combination therapy is also one of the excellent options for VD treatment.

Due to the lack of support from basic research, further studies are still needed to confirm the exact mechanism of action of BP, COPC, or VRC on VD. The small number of cases in this study and the short period may bias the results and tentatively prevent assessing the long-term prognostic impact of the two treatments on patients. We will conduct additional experiments to address the above limitations as soon as possible to provide a more reliable clinical reference.

## Conclusion

BP combined with COPC or VRC was effective in improving dementia symptoms, inducing neurological, cognitive, and hemodynamic recovery, and inhibiting inflammatory response and oxidative stress in VD patients after CI. Compared with BP combined with VRC, BP combined with COPC has a better therapeutic effect on patients and is more recommended for clinical use.

## Dodatak

### Ethical approval

The study protocol was approved by the Ethics Committee of The First Affiliated Hospital of Jiangxi Medical College (No. A202251).

### Availability of data and materials

The data supporting this study’s findings are available from the corresponding author upon reasonable request.

### Funding

The authors declare that no funds, grants, or other support were received during the. No funds, grants, or other support was received.

### Acknowledgements

Not applicable.

### Conflict of interest statement

All the authors declare that they have no conflict of interest in this work.
